# SpiDec: Computing binodals and interfacial tension of biomolecular condensates from simulations of spinodal decomposition

**DOI:** 10.3389/fmolb.2022.1021939

**Published:** 2022-10-24

**Authors:** Konstantinos Mazarakos, Ramesh Prasad, Huan-Xiang Zhou

**Affiliations:** ^1^ Department of Physics, University of Illinois at Chicago, Chicago, IL, United States; ^2^ Department of Chemistry, University of Illinois at Chicago, Chicago, IL, United States

**Keywords:** phase separation, biomolecular condensates, phase equilibrium, binodal, interfacial tension, spinodal decomposition

## Abstract

Phase separation of intrinsically disordered proteins (IDPs) is a phenomenon associated with many essential cellular processes, but a robust method to compute the binodal from molecular dynamics simulations of IDPs modeled at the all-atom level in explicit solvent is still elusive, due to the difficulty in preparing a suitable initial dense configuration and in achieving phase equilibration. Here we present SpiDec as such a method, based on spontaneous phase separation *via* spinodal decomposition that produces a dense slab when the system is initiated at a homogeneous, low density. After illustrating the method on four model systems, we apply SpiDec to a tetrapeptide modeled at the all-atom level and solvated in TIP3P water. The concentrations in the dense and dilute phases agree qualitatively with experimental results and point to binodals as a sensitive property for force-field parameterization. SpiDec may prove useful for the accurate determination of the phase equilibrium of IDPs.

## Introduction

**FIGURE 1:**
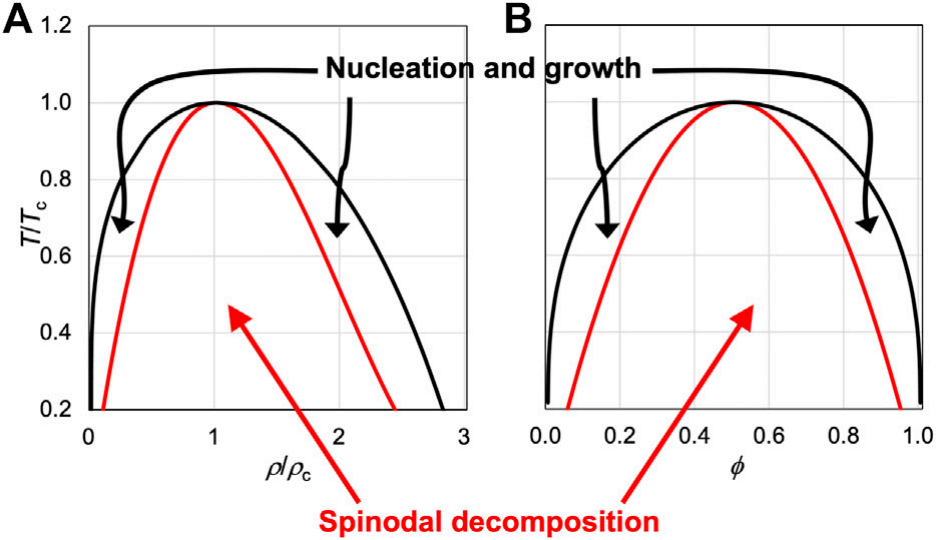
Binodals and spinodals of two model systems. **(A)** Van der Waals fluid, which satisfies the following equation of state: $\left(P+\frac{9}{8}k_{B}T_{c}\frac{\rho^{2}}{\rho_{c}}\right)\left(1-\frac{\rho }{3\rho_{c}}\right)=\rho k_{B}T$ , where P , ρ , T denote the pressure, density, and temperature, $T_{c}$ and $\rho_{c}$ denote the critical temperature and critical density, and $k_{B}$ is the Boltzmann constant. **(B)** Symmetric polymer blend that follows the Flory-Huggins theory for the Helmholtz free energy: $\frac{F}{Mk_{B}T}=\frac{\phi }{L}\ln\phi +\frac{1-\phi }{L}\ln\left(1-\phi \right)+\chi \phi \left(1-\phi \right)$ , where M is the total number of polymer chains, L is the number of beads per chain, ϕ is the mole fraction of one polymer species in the binary blend, and χ measures the energy gap between inter- and intra-species interactions. Inside the spinodal region, the system is unstable and phase separates by spinodal decomposition; between the binodal and spinodal, the system is metastable and phase separates by nucleation and growth. Note that there has been some controversy regarding the location of the spinodal in computer simulations (Binder et al., 2012; Díaz-Herrera et al., 2022).

Biomolecular condensates formed *via* liquid-liquid phase separation drive much of biology, but accurate computation of the binodal, representing the equilibrium concentrations in the bulk and dense phases as a function of temperature, based on atomistic modeling of the components presents a significant challenge. A related issue is the mechanism leading to phase separation. Theories predict that, depending on the initial densities or compositions, phase separation occurs by two mechanisms (Figure 1). Inside the spinodal, the system is thermodynamically unstable and phase separation occurs spontaneously, in a process known as spinodal decomposition. This process is initiated by large-scale density fluctuations, leading to interconnected domains. Further condensation then leads to separated condensates. Between the binodal and spinodal, the system is metastable, and phase separation occurs by nucleation and growth. This mechanism is initiated by local density fluctuations, leading to the generation of nuclei. Further growth then produces stable condensates. The dividing line between these two mechanisms, i.e., the spinodal, is known for a number of theoretical models (Figure 1) and has been measured for both structured and disordered proteins (Thomson et al., 1987; Bracha et al., 2018). Both spinodal decomposition and nucleation have been observed for the formation of biomolecular condensates (Bracha et al., 2018; Kasinsky et al., 2021). It has been suggested that cells may want to keep component concentrations to a minimum required for phase separation, i.e., crossing the low-concentration branch of the binodal just enough into the left metastable region, and thus nucleation and growth is the favored mechanism (Zeng et al., 2020). However, in most theoretical models (Figure 1), the spinodal covers the bulk of the area under the binodal, and therefore random initial preparations have much higher chances for phase separation by spinodal decomposition than by nucleation and growth. Indeed, phase separation is observed instantaneously in many *in vitro* preparations; the rapid speed is consistent with spinodal decomposition.

In computer simulations of model systems, depending on the initial density, spinodal decomposition produces a variety of morphologies for the dense phase, including sphere, cylinder, slab, hollow cylinder, and hollow sphere (Binder et al., 2012; Díaz-Herrera et al., 2022). The morphologies are a reflection of the finite system size, where surface energy associated with interfacial tension becomes nonnegligible compared to the free energy within the dense phase. In essence, each shape optimizes the balance between these two terms of free energy at the given density. The slab morphology is of special interest because it represents the macroscopic form of the dense phase. More importantly, for this paper, the slab morphology is the basis of the method for computing binodals to be presented below.

Over the years, a variety of methods have been developed to compute the binodals of coarse-grained model systems (for a brief review and illustration, see Mazarakos et al., 2023). One of the earliest methods is based on preparing a dense slab at the center of an otherwise empty simulation box (Rao and Levesque, 1976). Molecular dynamics (MD) simulations then allow the dense and bulk phases to reach equilibration, and the densities of the two phases are then evaluated to build the binodal. This classical slab method has now been applied to a variety of coarse-grained models of biomolecular condensates, from spherical particles and homopolymers (as crude models of structured proteins and IDPs) (Mazarakos and Zhou, 2021; Mazarakos and Zhou, 2022) to residue-level models of IDPs (Das et al., 2018; Dignon et al., 2018; Statt et al., 2020) to multi-bead-per-residue models of dipeptides (Tang et al., 2021). An exciting recent development is the migration of this method to simulations of all-atom models of IDPs in explicit solvent, by first carrying out full simulations at the coarse-grained level and then mapping to atomistic systems (Zheng et al., 2020; Welsh et al., 2022). Still, the mapping is not a trivial task and, after mapping, simulations at the all-atom level may fail to allow exchange of protein molecules between the phases, let alone reaching phase equilibrium (Zheng et al., 2020). In comparison, Gibbs ensemble simulations (Panagiotopoulos, 1987), while proven useful for providing insights on biomolecular condensates (Nguemaha and Zhou, 2018; Ghosh et al., 2019) and complex coacervates (Lytle et al., 2016; Li et al., 2018), are more restrictive, in particular due to the difficulty in inserting a polymer chain into a dense solution. Recent implementation by field-theoretic simulations has widened the applicability of the Gibbs ensemble (McCarty et al., 2019), but application to all-atom models seems out of reach at present. Lastly the FMAP method, based on using the fast Fourier transform to evaluate intermolecular interaction energies, has been developed to calculate the binodals of structured proteins, but not IDPs, modeled at the all-atom level in implicit solvent (Qin and Zhou, 2016).

Here we present a method that we dub SpiDec, as an efficient alternative to the classical slab method. Instead of an initial dense slab, we start the system at a homogeneous, low density inside the spinodal. Spinodal decomposition then quickly brings the system to a slab morphology. We demonstrate this method both on four model systems and on a phase-separating tetrapeptide (Abbas et al., 2021) at the all-atom level in explicit solvent.

## Computational methods

### Model systems

We tested the SpiDec method on model systems composed of Lennard-Jones (LJ) particles, LJ chains, hydrophobic-hydrophilic (HP) chains, and patchy particles. The interaction potentials for LJ particles and LJ chains are the same as in our previous studies (Mazarakos and Zhou, 2021; Mazarakos and Zhou, 2022). Specifically, the LJ particles interact *via* the LJ potential,

$$U_{LJ}\left(r\right)=4\varepsilon \left[{\left(\frac{\sigma }{r}\right)}^{12}-{\left(\frac{\sigma }{r}\right)}^{6}\right]$$ (1)

but with a cutoff imposed at $r_{c}=3\sigma$ , so the actual potential function is shifted to

$$U_{LJ}^{S}\left(r\right)=\left\{ \begin{array}{c} U_{LJ}\left(r\right)-U_{LJ}\left(r_{c}\right),\;r<r_{c}\\ 0,\;r\geq r_{c} \end{array}\right.$$ (2)

The LJ chains consist of 10 beads. The beads interact *via* a force-shifted LJ potential:

$$U_{LJ}^{FS}\left(r\right)=\left\{ \begin{array}{c} U_{LJ}\left(r\right)-U_{LJ}\left(r_{c}\right)-{\frac{dU_{LJ}\left(r\right)}{dr}|}_{r=r_{c}}\left(r-r_{c}\right),\;r<r_{c}\\ 0,\;r\geq r_{c} \end{array}\right.$$ (3)

with a cutoff $r_{c}=6\sigma$ . This potential is not applied to adjacent beads in a chain; instead they are connected by harmonic bonds with an equilibrium length of σ and a spring constant of 75,000 $\varepsilon /\sigma^{2}$ . Some simulations were also carried out for the LJ chains with the spring constant reduced to 750 $\varepsilon /\sigma^{2}$ and 75 $\varepsilon /\sigma^{2}$ . The HP chains are very much like the LJ chains, except that there are two kinds of beads, H and P (Statt et al., 2020). Each chain has two P beads, at positions 1 and 5; the rest are H beads. Some simulations were also carried out with the two P beads at positions 5 and 6. H beads interact with each other *via* the above force-shifted LJ potential, whereas all other pairwise interactions (H-P and P-P) were the purely repulsive Weeks-Chandler-Anderson (WCA) potential (Weeks et al., 1971),

$$U_{WCA}\left(r\right)=\left\{ \begin{array}{c} U_{LJ}\left(r\right)+\varepsilon ,\;r<2^{1/6}\sigma \\ 0,\;r\geq 2^{1/6}\sigma \end{array}\right.$$ (4)

The interaction potential for patchy particles was the same as in our previous studies (Nguemaha and Zhou, 2018; Ghosh et al., 2019). Each particle has four equal-sized circular patches with centers located at the vertices of a tetrahedron. Each patch has a spanning polar angle of $\theta_{s}$ , which is set to a value ( $\cos\theta_{s}$ = 0.35) so the patches cover a fraction of 0.7 of the particle surface. The particles have a diameter σ and interact *via* the potential (Kern and Frenkel, 2003).

$$U_{PP}\left(r,\Omega_{1},\Omega_{2}\right)=\left\{ \begin{array}{r} \infty ,\;r<\sigma \\ -\varepsilon \underset{\alpha \beta }{\sum }f_{\alpha \beta }\left(\hat{r},\Omega_{1},\Omega_{2}\right),\;\sigma \leq r<\sigma +\lambda \\ 0,\;r\geq \sigma +\lambda \end{array}\right.$$ (5)

where the range of interaction, *λ*, is fixed to 0.5*σ*; $\hat{r}$ denotes the unit vector along the inter-particle displacement r (pointing from particle 1 to particle 2); $\Omega_{i}$ denotes the orientation of particle *i*; and $f_{\alpha \beta }\left(\hat{r},\Omega_{1},\Omega_{2}\right)$ is 1 if patch α of particle 1 is “bonded” with patch β of particle 2, and 0 otherwise. The bonding condition is satisfied if the inter-particle vector falls within both patches, i.e., $\hat{r}∙n_{1\alpha }>\cos\theta_{s}$ and $-\hat{r}∙n_{2\beta }>\cos\theta_{s}$ , where $n_{i\alpha }$ is the unit vector from the center of particle *i* to the center of patch α on particle *i*. The orientation of each patchy particle was specified by the unit vectors along the three axes of a Cartesian coordinate system fixed to the particle.

### Initialization for simulations

We studied LJ particles by both MD and Monte-Carlo (MC) simulations, LJ and HP chains by MD simulations, and patchy particles by MC simulation. The simulation boxes were rectangular, with side length *L*_*x*_ in two directions and *L*_*z*_ (≥*L*_*x*_) in the third direction. To prepare for MD simulations, particles or chains were randomly inserted into the simulation box at density *ρ*_0_. For MC simulations, the particles were initially placed on a cubic lattice spanning the simulation box, again at an initial density *ρ*_0_. The values of *ρ*_0_ are given below. The periodic boundary condition was applied during the simulations.

### Ranges in initial density for various dense-phase morphologies

We scanned the initial density to identify the dense-phase morphologies of a given system, with the temperature at the lowest value (0.65, 1.70, 1.05, and 0.61 for LJ particles, LJ chains, HP chains, and patchy particles, respectively). The initial density was scanned up to 0.8, in two series. In the first series, the particle numbers were fixed (1,000 for LJ particles and LJ and HP chains; 750 for patchy particles); the simulation boxes were cubic, and the side lengths were varied to span the range of initial densities, which was from 0.05 to 0.8 at increments of 0.05.

In the second series, done for LJ particles and LJ chains, *L*_*x*_ was fixed (10 for LJ particles and 13 for LJ chains), and *L*_*z*_/*L*_*x*_ was varied from 1 to 5 in increments of 0.25 or 0.5. The initial densities were 0.01–0.12 in increments of 0.01, 0.125 to 0.3 in increments of 0.025, 0.3 to 0.8 (for LJ particles) or 0.7 (for LJ chains) in increments of 0.05. The corresponding particle (or bead) numbers ranged from 10 to 4,000 for LJ particles and from 20 to 7,690 for LJ chains. For a given *L*_*z*_/*L*_*x*_, at increasing initial densities, spinodal decomposition or lack thereof results in seven distinct cases: a low-density homogenous phase, five dense-phase morphologies, and a high-density homogenous phase. For the boundary between the case of a low-density homogenous phase and the case of a dense phase with a spherical morphology, we took the midpoint between the highest initial density that resulted in a low-density homogenous phase and the lowest initial density that resulted in a dense phase with a spherical morphology. Sometimes it was uncertain whether to identify the status resulted from an intermediate initial density as a low-density homogenous phase or a dense phase with a spherical morphology; we then assigned that initial density as the boundary value. A similar procedure was followed to identify the next boundary, i.e., between a dense phase with a spherical morphology and a dense phase with a cylindrical morphology. The process continued until the last boundary, i.e., between a dense phase with a hollow sphere and a high-density homogenous phase, was determined.

### Molecular dynamics simulations for LJ particles and LJ and HP chains

These simulations were carried out using the HOOMD-blue package (version 2.5.0) on GPUs (Glaser et al., 2015). All the particles or beads have the same diameter σ , which sets the unit of lengths, and the same mass m . The units for number density, temperature, interfacial tension, and time are $\sigma^{-3}$ , $\varepsilon /k_{B}$ , $\varepsilon /\sigma^{2}$ , and $\sqrt{m\sigma^{2}/\varepsilon }\equiv \tau$ , respectively. Below we will often leave out these units in order to reduce clutter. The MD simulations were carried out at constant particle (or bead) number (*N*), volume, and temperature. Temperature was regulated by the Langevin thermostat with a friction coefficient of 0.1 m/τ . The time step was 0.005 τ for particle systems and 0.001 τ for chain systems. For identifying the dense-phase morphologies of a given system, the initial densities were scanned over a range as described above. The presentation below applies to simulations where a slab morphology was formed and used to calculate binodals and interfacial tension.

The initial densities were 0.3 for LJ particles and 0.25 for LJ and HP chains. Separate simulations were carried out over a range of temperatures. To investigate the effects of system size and *L*_*z*_/*L*_*x*_ ratio, we chose *N* in the range of 1,000–10,000, and *L*_*z*_/*L*_*x*_ from 1.25 to 33.3 for LJ particles, from 1.16 to 18.2 for LJ chains, and 1.5 to 5 for HP chains. For LJ particles, the simulation length was 10 million steps, but was extended to 100 or 200 million steps when multiple slabs took a long time to fuse into a single slab (at *N* ≥ 6,000 and *L*_*z*_/*L*_*x*_ ≥ 20). The same was true for LJ chains, except that the longer simulations were 100–300 million steps and applied to more cases (*N* as low as 4,000 and *L*_*z*_/*L*_*x*_ as low as 2). The simulation length was 100 million steps for HP chains. The time interval for saving snapshots was 1,000 time steps for simulations with a total length of 10 million steps and 10,000 time steps for longer simulations.

### Monte Carlo simulations

For patchy particles, the initial density was 0.36, *N* ranged from 250 to 1,250, and *L*_*z*_/*L*_*x*_ was 1.5, 3, and 5. MC simulations were run for 2 million steps, and up to 5 million steps for larger *N* and elongated simulation boxes. Snapshots were saved for analysis once every 2000 MC steps. Each MC step consisted of either a displacement or a rotation (with equal probability) for every particle. The displacement was randomly selected inside a cube centered at the original position and with a side length of 0.09 σ . The rotation was realized by picking an arbitrary new direction for the *z* axis of the particle-fixed coordinate system and rotating the line of nodes, defined as the cross product of the old and new *z* axes, by an angle to become the new *x* axis of the particle-fixed coordinate system (Nguemaha and Zhou, 2018). The latter was arbitrarily chosen between −0.05 and 0.05 radians. An MC simulation was also carried out for LJ particles at *ρ*_0_ = 0.3, *N* = 1,000, and *L*_*z*_/*L*_*x*_ = 3 for 3 million steps (saving every 100 MC steps) to determine the binodal and interfacial tension, for comparison with the results obtained by MD simulations. In this case no rotation was included in the MC step.

### Time for phase separation *via* spinodal decomposition

In simulations where the dense phase had a slab morphology, we monitored the time that it took for slabs to emerge from spinodal decomposition. In each saved snapshot, the density profile along the normal direction of the slabs (typically the *z* direction) was calculated in slices with a default thickness of 1 σ and the maximum density was collected as a function of simulation time. After slabs were formed, the maximum densities reached a plateau. We took the first time that the maximum density exceeded the plateau value as the time to phase separate, denoted by $\tau_{PS}$ . In some cases the default thickness of the slices for density calculation was adjusted so the resulting $\tau_{PS}$ was close to the value obtained by visual inspection, in which we looked for slabs with relatively flat surfaces.

When multiple slabs were formed, we also monitored the time, $\tau_{SS}$ , that it took for the slabs to fuse one by one, finally into a single slab. The number of slabs in each saved snapshot after $\tau_{PS}$ was determined by the following procedure. Again, the density profile was calculated in slices of thickness 1 σ . Each slice was then labeled as “H” (for high density) if its density exceeded a high cutoff *ρ*_H_, as “L” (for low density) if its density dipped below a low cutoff *ρ*_L_, or filtered out if its density fell between *ρ*_H_ and *ρ*_L_. In this way the density profile was converted to a sequence like HHHHLLL…HHH. Each transition from H to L or L to H in this sequence defined an interface. The number of slabs was 1/2 of the number of interfaces. The first time that the number of slabs reduced to 1 was $\tau_{SS}$ . The high cutoff density was in the range of 0.5–0.6 whereas the low cutoff density was in the range of 0.1–0.4; their precise values were selected for each system so the resulting $\tau_{SS}$ was close to the value obtained by visual inspection, in which we looked for a single slab in the entire simulation box.

### Densities in the dense and bulk phases

In all the simulations where a slab morphology was formed and used to calculate equilibrium properties, averages were taken over the second half of the simulations. However, when $\tau_{SS}$ occurred in the second half of a simulation, as in a few cases for LJ chains and more cases for patchy particles with large *N* and long boxes, averages were only taken from $\tau_{SS}$ to the end of the simulation, to ensure that the simulation box contained only a single slab in this calculations.

The density profile, ρ(z) , along the *z* direction was calculated by dividing the simulation box into slices of thickness 0.1 σ along *z*. In each snapshot, the periodic system was translated to have the center of mass located at the center of the simulation box. The total number of particles (or beads) in each slice was then divided by its volume to yield an estimate for the density at that particular *z* in that snapshot. This estimate was then averaged over all the snapshots saved for analysis to obtain ρ(z) . In cases where the slab was oriented with its normal along *x* or *y* (occurring only when *L*_*z*_/*L*_*x*_ was close to 1), the density profile was calculated along that direction.

To obtain the densities, $\rho_{d}$ and $\rho_{b}$ , in the dense and bulk phases, we fit the density profile in the positive *z* range to the following function:

$$\rho (z)=\frac{\rho_{d}+\rho_{b}}{2}-\frac{\rho_{d}-\rho_{b}}{2}\tanh\left[(z-z_{0})/w\right]$$ (6)

where $z_{0}$ represents the midpoint of the interface between the two phases, and w is a measure of the width of the interface.

### Critical temperature

The binodal, comprising bulk- and dense-phase densities as a function of temperature, was fit to the following equations

$$\frac{1}{2}\left(\rho_{b}+\rho_{d}\right)=\rho_{c}+A\left(T-T_{c}\right)$$ (7a)

$$\rho_{d}-\rho_{b}=B{\left(T_{c}-T\right)}^{\beta }$$ (7b)

where $T_{c}$ is the critical temperature, $\rho_{c}$ is the critical density, A and B are constants, and the exponent β is set to 0.32.

### Interfacial tension

The interfacial tension γ was determined according to the Kirkwood-Buff method (Kirkwood and Buff, 1949):

$$\gamma =\frac{L_{z}}{2}\langle p_{zz}-\frac{p_{xx}+p_{yy}}{2}\rangle$$ (8)

where $p_{xx}$ , $p_{yy}$ , and $p_{yy}$ are the diagonal elements of the pressure tensor, and the brackets indicate an equilibrium average. We calculated these diagonal elements on snapshots separated by 10 time steps for the MD simulations and averaged them over the same portion of each simulation as used for calculating densities. Interfacial tension was not calculated for patchy particles, because the pressure tensor could not be properly defined due to the discontinuous nature of the interaction potential. Interfacial tension was determined for LJ particles from an MC simulation, from pressure tensor calculated at every 100 MC steps and averaged over the second half of the simulation.

### Molecular dynamics simulations of phase-separating tetrapeptide

We also tested SpiDec on a peptide, consisting of two copies of a phenylalanine dipeptide crosslinked at the C-termini by a disulfide bond (denoted as FFssFF), that was recently shown to phase separate (Abbas et al., 2021). FFssFF was prepared in ChemDraw and saved in Protein Data Bank (PDB) format for force-field parametrization, which was done using Gaussian 16 at the HF/6-31G* level for atomic charges and using general Amber force field (GAFF) (Wang et al., 2004) for other parameters. The initial configuration of 64 copies of the peptide in a water box was prepared in two steps. First, eight copies were randomly inserted into a cubic box with a side length of 30 Å and solvated with TIP3P water (Jorgensen et al., 1983) using CHARMM-GUI (Jo et al., 2008). This system was relaxed by energy minimization (2000 steps of steepest descent and 3,000 steps of conjugate gradient) and a 100 ps MD simulation at constant NVT (ramping from 0 to 294 K in 40 ps and at 294 K for remaining 60 ps) with a 1 fs timestep. The box with only the peptides in the last snapshot was duplicated in each of the three orthogonal directions to build a system with 64 copies in a cubic box with a side length of 60 Å, which was solvated again with 4693 TIP3P water molecules. The latter system was also relaxed by energy minimization and 500 ps of constant-NVT simulation (ramping from 0 to 294 K in 40 ps and at 294 K for remaining 460 ps). To further condense the system, half of the water molecules were randomly removed and the system was again relaxed by the same procedure of energy minimization plus 500 ps of constant-NVT simulation. It was then equilibrated at constant NPT (294 K and 1 bar) and with a 2 fs timestep, for 8.6 µs until the peptides formed a single slab with normal in the *z* direction. The side length of the cubic box at this point was reduced to 51.72 Å. Long-range electrostatic interactions were treated by the particle mesh Ewald method (Essmann et al., 1995) with nonbonded cutoff at 8 Å. Temperature was regulated by the Langevin thermostat with a damping constant of 3 ps^−1^; pressure was regulated using the Berendsen barostat (Berendsen et al., 1984). All bonds connected with hydrogen atoms were constrained using the SHAKE algorithm (Ryckaert et al., 1977).

The single slab of 64 copies was finally placed in a rectangular box with *L*_*z*_/*L*_*x*_ = 5. To model pH 7, 32 copies were randomly chosen to have a terminal amide protonated. The system was neutralized with 32 Cl^−^ ions and solvated with 20,174 TIP3P water molecules; the total number of atoms was 66,986. Initial relaxation was done with energy minimization and 500 ps of constant NVT simulation with a 1 fs timestep, followed by 100 ns of equilibration at constant NPT with a 2 fs timestep. Finally constant-NVT production runs with a 2 fs timestep, at *T* = 294 K and 326 K, each for 2 µs. The box dimensions were 51.47 Å × 51.47 Å × 260.38 Å and 52.04 Å × 52.04 Å × 263.24 Å, respectively, at the two temperatures. The last snapshot of the 326 K simulation was ramped to 340 K or 360 K (in a 500 ps of constant NVT simulation) and production runs were carried out at these higher temperatures for 2 µs each. All MD simulations were carried out on GPUs using *pmemd.cuda* (Salomon-Ferrer et al., 2013). Snapshots were saved at 100 ps intervals for analysis.

## Results

### Variety of dense-phase morphologies from spinodal decomposition

**FIGURE 2:**
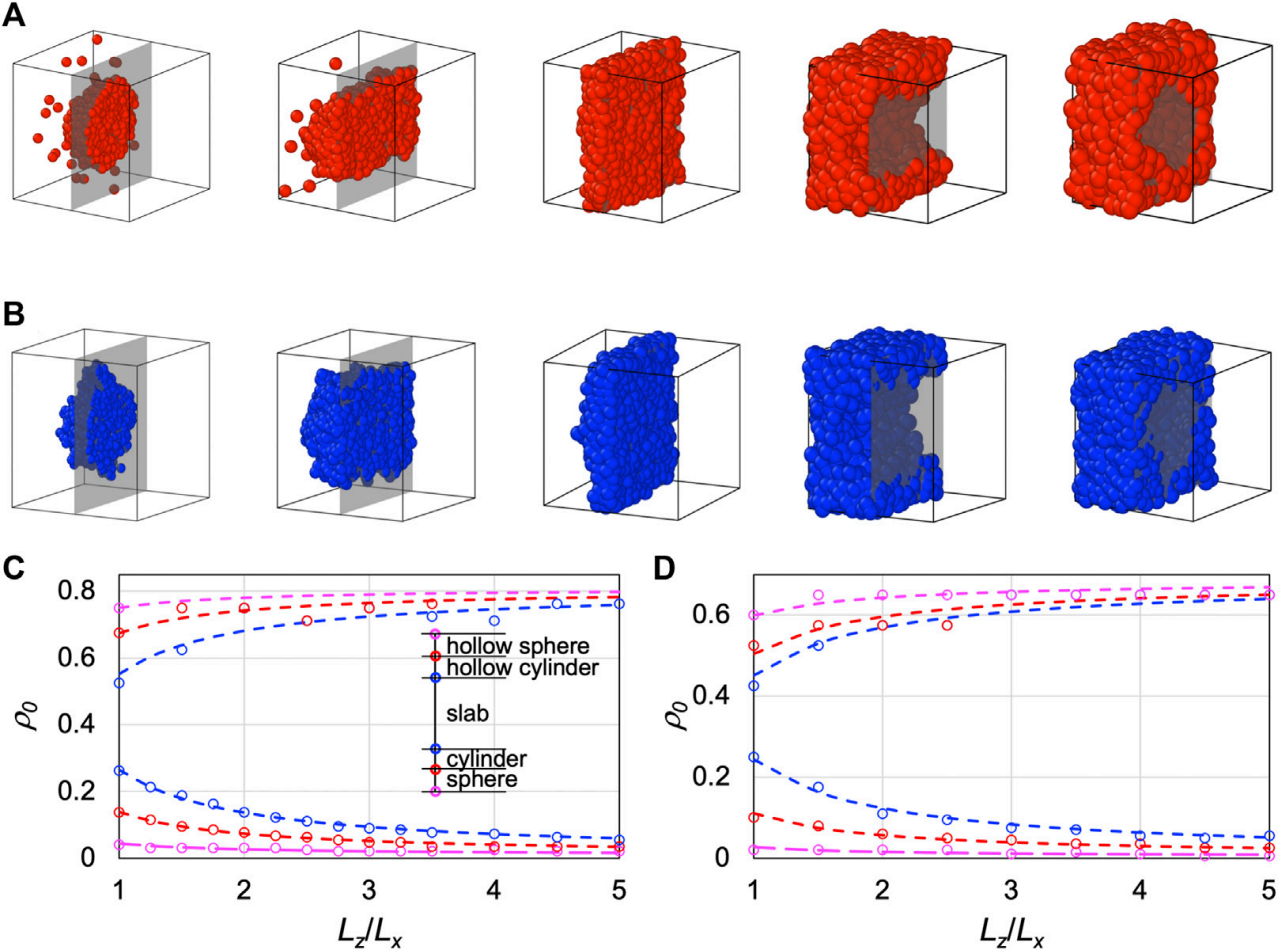
Morphologies of the dense phases of LJ particles and LJ chains over a range of initial densities inside the spinodal. **(A)** Morphologies for an LJ particle system of *N* = 1,000 particles in a cubic box at *T* = 0.65. The dense phase appears as a sphere, cylinder, slab, hollow cylinder, and hollow sphere at *ρ*_0_ = 0.1, 0.2, 0.3, 0.6, and 0.7 respectively. The initial densities were changed by varying the box side lengths, which are shown here not to scale. At each density, the cubic box is cut by a plane (rendered as gray when the background is empty), and only the half behind the cut is displayed. **(B)** Corresponding results for an LJ chain system (100 10-bead chains) at *T* = 1.7 and *ρ*_0_ = 0.1, 0.2, 0.3, 0.5, and 0.6. **(C)** Boundaries between different morphologies for LJ particles in rectangular boxes with different *L*_*z*_/*L*_*x*_ ratios. The density ranges for different morphologies are illustrated in the inset. *L*_*x*_ = 10 and *T* = 0.65. **(D)** Corresponding results for an LJ chain system at *L*_*x*_ = 13 and *T* = 1.7.

In Figure 2A, we display the dense-phase morphologies of LJ particles in a cubic box at *T* = 0.65, obtained from MD simulations at increasing initial densities (*ρ*_0_). The dense phase appears as a sphere at *ρ*_0_ = 0.1, a cylinder at *ρ*_0_ = 0.2, a slab at *ρ*_0_ = 0.3, a hollow cylinder at *ρ*_0_ = 0.6, and a hollow sphere at *ρ*_0_ = 0.7. Similar observations are found for the other model systems, and are displayed in Figure 2B for LJ chains at *T* = 1.7 and in Supplementary Figure S1 for HP chains at *T* = 1.05.

We scanned the initial densities to identify the boundaries between different dense-phase morphologies. For example, for LJ particles in a cubic box (*L*_*z*_/*L*_*x*_ = 1) at *T* = 0.65, the transitions from a single low-density phase to a spherical dense phase, from sphere to cylinder, from cylinder to slab, from slab to hollow cylinder, from hollow cylinder to hollow sphere, and from hollow sphere to a single high-density phase occur at initial densities of 0.04, 0.1375, 0.2625, 0.525, 0.675, and 0.75, respectively. Note that we checked the dense-phase morphologies on the very short timescale of phase separation *via* spinodal decomposition (see below for more details). On this timescale, phase separation *via* nucleation and growth would not have occurred. Therefore simulations from initial densities in the metastable regions, between the spinodal and binodal, would remain a single phase, and the boundaries with the two single phases effectively define the spinodal densities. In the foregoing example, the lowest and highest boundary values, 0.04 and 0.75, are the spinodal densities.

At increasing *L*_*z*_/*L*_*x*_, the range of initial densities for the slab morphology widens in both directions, squeezing the other inter-morphology boundaries at both ends of the range (Figure 2C). The dependences of the boundary density values on *L*_*z*_/*L*_*x*_ fit well to a simple function,

$$\rho_{0}=\frac{\rho_{1}+\rho_{\infty }\xi }{1+\xi }$$ (9)

where $\xi =L_{z}/L_{x}-1$ , $\rho_{1}$ denotes the density at $L_{z}/L_{x}$ = 1, and $\rho_{\infty }$ represents the density extrapolated to infinite $L_{z}/L_{x}$ . The three lower boundaries have a common $\rho_{\infty }$ value of 0.015, whereas the three upper boundaries have a common $\rho_{\infty }$ value of 0.81. Similar observations are found for the other model systems. The results for LJ chains at *T* = 1.7 are shown in Figure 2D, where the fit to Eq. 9 yields a $\rho_{\infty }$ value of 0.006 for the three lower branches and a $\rho_{\infty }$ value of 0.69 for the three upper branches. These lower and upper $\rho_{\infty }$ values may represent the spinodal densities at infinite system sizes.

### Slab formation at different *L*_*z*_/*L*_*x*_ ratios

**FIGURE 3:**
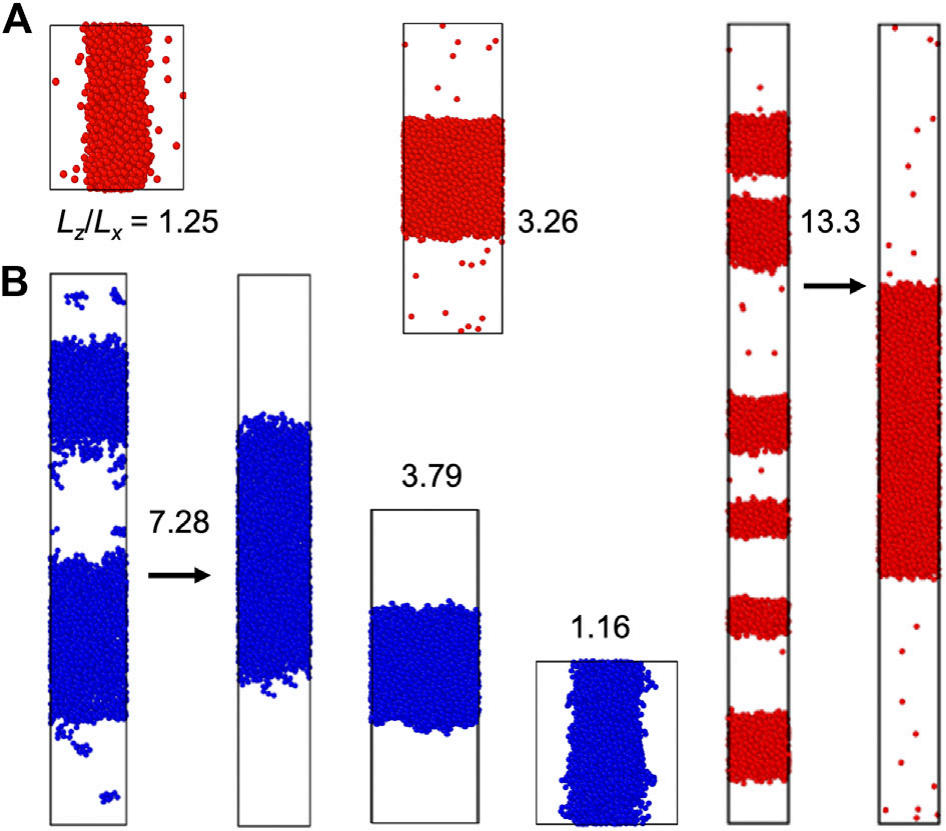
Slab formation at various *L*_*z*_/*L*_*x*_ values. **(A)** LJ particle system at *N* = 4,000, *T* = 0.65, *ρ*_0_ = 0.3, and *L*_*z*_/*L*_*x*_ = 1.25, 3.26, and 13.3. **(B)** LJ chain system at *N* = 4,000, *T* = 1.7, *ρ*_0_ = 0.25, and *L*_*z*_/*L*_*x*_ = 1.16, 3.79, and 7.28. The *z* axis is along the vertical direction. For each system, the simulation boxes are drawn approximately to scale. Arrows indicate the fusion of multiple slabs into a single slab.

**FIGURE 4:**
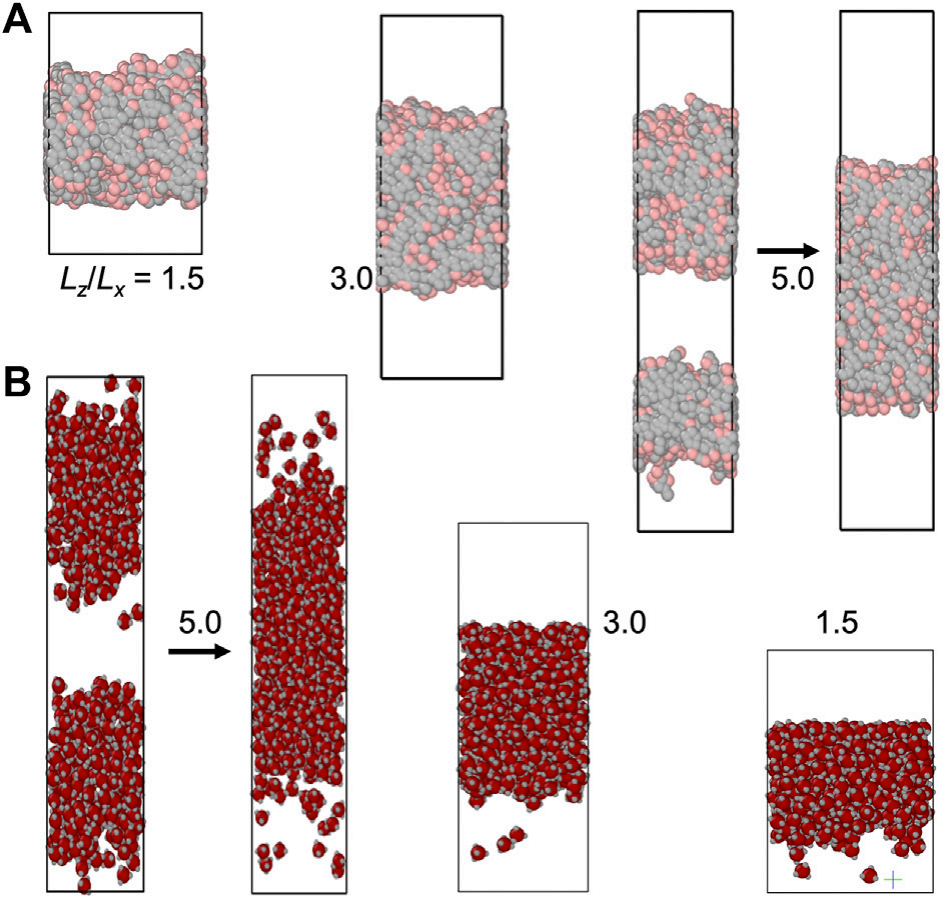
Slab formation at various *L*_*z*_/*L*_*x*_ values. **(A)** HP chain system at *N* = 4,000, *T* = 1.05, *ρ*_0_ = 0.25, and *L*_*z*_/*L*_*x*_ = 1.5, 3.0, and 5.0. **(B)** Patchy particle system at *N* = 500, *T* = 0.61, *ρ*_0_ = 0.36, and *L*_*z*_/*L*_*x*_ = 1.5, 3.0, and 5.0. The *z* axis is along the vertical direction. For each system, the simulation boxes are drawn approximately to scale. Arrows indicate the fusion of multiple slabs into a single slab.

The slab morphology allows easy determination of binodals (see Computational Methods). We thus pay special attention to this morphology. In Figures 3, 4, we display snapshots of slabs formed in simulations of the four model systems over a range of $L_{z}/L_{x}$ . In a cubic simulation box, there is no preferred direction, and so slabs can form with the normal along any of the three orthogonal directions. The indeterminacy in slab orientation persists when $L_{z}/L_{x}$ is slightly above 1 (Figures 3A,B). Regardless of the slab orientation, the thickness of the bulk phase in simulation boxes with $L_{z}/L_{x}$ close to 1 is small, which would make it difficult to calculate the bulk-phase density and determine the binodal.

At $L_{z}/L_{x}$ above ∼2, slabs are always oriented with the normal along the *z* direction. As $L_{z}/L_{x}$ is further increased above ∼4, multiple slabs can form (Figures 3, 4). Over time these multiple slabs fuse one by one, eventually leading to the complete fusion into a single slab (Supplementary Movie S1).

### Timescales for phase separation *via* spinodal decomposition and for complete slab fusion

We devised a procedure to determine the time ( $\tau_{PS}$ ) at which slabs first emerge from spinodal decomposition (Supplementary Figure S2A). The results for three model systems from MD simulations at *N* = 4,000 are shown in Supplementary Figure S3A. For LJ particles, slab formation is quick, occurring on the order of 2 × 10^4^ time steps in MD simulations. $\tau_{PS}$ decreases with increasing $L_{z}/L_{x}$ . For LJ and HP chains, $\tau_{PS}$ shows an even stronger initial decline (near $L_{z}/L_{x}$ = 1). In the range of $L_{z}/L_{x}$ from 2 to 7, the $\tau_{PS}$ values (in LJ time units) for LJ and HP chains are 3–8 times as long as that for LJ particles. For patchy particles at *N* = 500 and $L_{z}/L_{x}$ from 1.5 to 5, $\tau_{PS}$ is of the order of 3 × 10^5^ MC steps.

For the purpose of determining binodals, another important timescale is $\tau_{SS}$ , for complete fusion into a single slab when multiple slabs emerge from spinodal decomposition (Supplementary Figure S2B). The results for $\tau_{SS}$ are shown in Supplementary Figure S3B. In contrast to $\tau_{PS}$ , $\tau_{SS}$ exhibits a sharp increasing trend with increasing $L_{z}/L_{x}$ . The main reason for the increase in $\tau_{SS}$ is that, with higher $L_{z}/L_{x}$ , more slabs form initially and hence more fusion events must take place before complete fusion. A secondary reason is that, with higher $L_{z}/L_{x}$ , slabs are farther apart initially and hence each fusion event can take longer. In any event, for LJ particles, $\tau_{SS}$ is under a million time steps even at $L_{z}/L_{x}$ = 13.3. Since we equilibrate at least 5 million time steps anyway, so slab fusion for LJ particles at *N* = 4,000 did not necessitate longer simulations. The same holds for LJ and HP chains at *N* = 4,000 and $L_{z}/L_{x}$ ≤ 5, with $\tau_{SS}$ under 3.1 million time steps. However, for LJ chains at $L_{z}/L_{x}$ = 7.28, $\tau_{SS}$ exceeds 5 million time steps, and as a result we extended the simulations to 200 million steps. For patchy particles at *N* = 500 and $L_{z}/L_{x}$ from 3 to 5, $\tau_{SS}$ is 0.9–1.8 million MC steps.

### Optimum in *L*_*z*_/*L*_*x*_ and selection of *ρ*_0_ for binodal determination

So it appears that there is an optimum in $L_{z}/L_{x}$ in performing simulations to form a single slab for binodal determination. When $L_{z}/L_{x}$ is too small (e.g., below 2), a single slab may directly form from spinodal decomposition, but the orientation of the slab is indeterminant and the thickness of the bulk phase in the simulation box may be too small for a precise determination of the bulk-phase density. On the other hand, when $L_{z}/L_{x}$ is too large (e.g., above 7), multiple slabs emerge from spinodal decomposition and can take a long time to fuse into a single slab. This long fusion time will increase the total simulation time. The optimal $L_{z}/L_{x}$ is from 3 to 5.

Slab formation also requires a correct choice for the initial density. Fortunately, at $L_{z}/L_{x}$ from 3 to 5, the range of initial densities leading to slab formation is pretty wide (see Figures 2C,D). Here is a general procedure for selecting an appropriate *ρ*_0_ to start the simulation. The procedure involves running short simulations (e.g., 1 million steps; see Supplementary Figure S3A) at an $L_{z}/L_{x}$ between 3 and 5 and *ρ*_0_ at $i\Delta \rho_{0}$ , with i = 1, 2, …, and $\Delta \rho_{0}$ between 0.05 and 0.1. As *ρ*_0_ is increased, a dense phase should emerge, with shapes in the order of sphere, cylinder, slab, hollow cylinder. Finally choose the midpoint in the range of initial densities leading to slab formation. This procedure can be applied once, at the lowest temperature for which the binodal is to be determined. Once the initial density is found at this temperature, it can be used for long simulations in the full temperature range to determine the binodal.

### Binodals of four model systems

**FIGURE 5:**
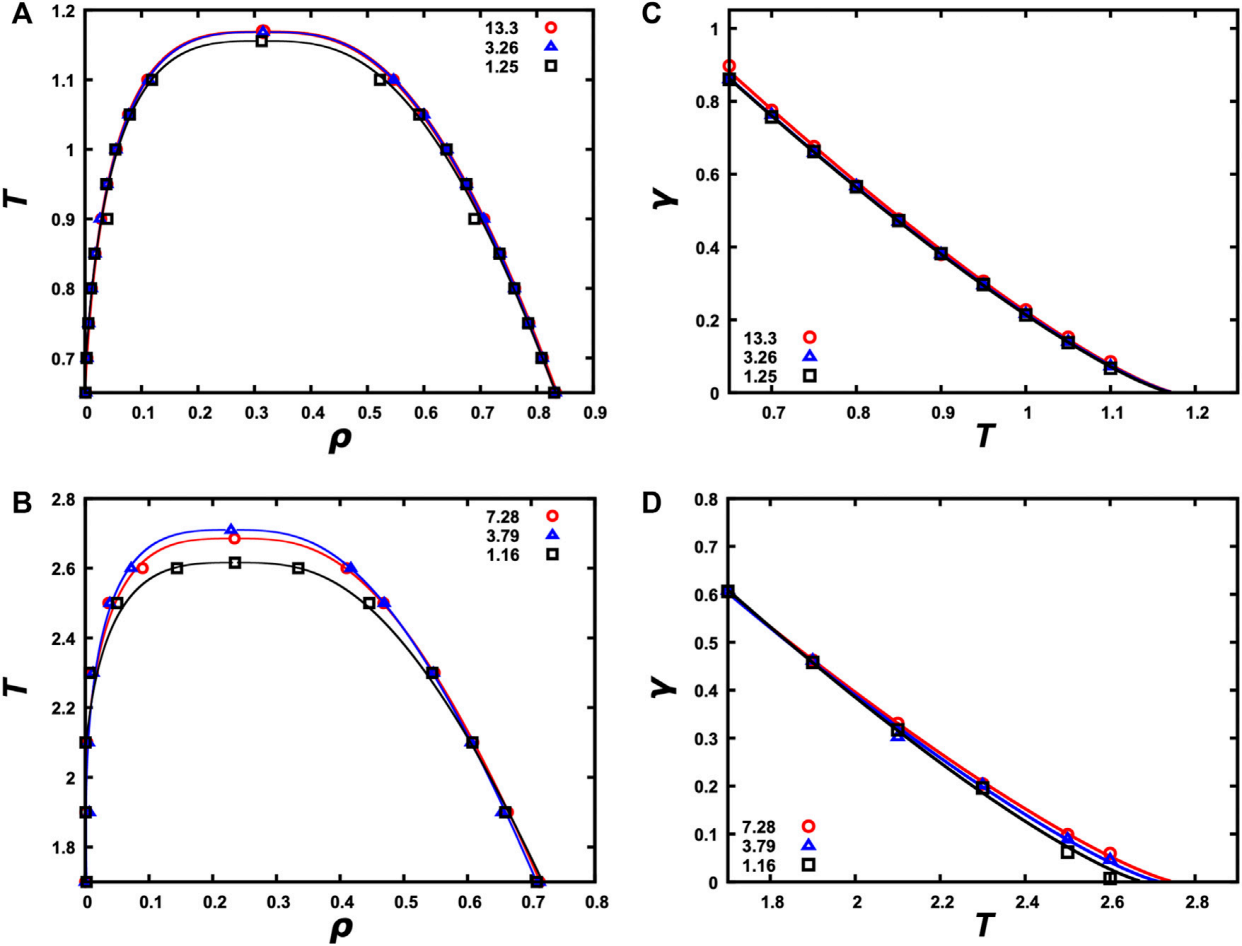
Binodals and interfacial tensions calculated from snapshots with a single slab. **(A)** Binodals of the LJ particle system at *N* = 4,000 and *L*_*z*_/*L*_*x*_ = 1.25, 3.26, and 13.3. **(B)** Binodals of the LJ chain system at *N* = 4,000 and *L*_*z*_/*L*_*x*_ = 1.16, 3.79, and 7.28. **(C)** Interfacial tension *versus* temperature for the LJ particle system at the three *L*_z_/*L*_*x*_ ratios. **(D)** Interfacial tension *versus* temperature for LJ chain system at the three *L*_z_/*L*_*x*_ ratios.

Once a single slab is formed and equilibrated with the bulk phase at each temperature within a selected range, we can calculate the densities of the two phases as a function of temperature. The resulting binodals are shown in Figures 5A,B for the LJ particle and LJ chain systems at *N* = 4,000 and in Supplementary Figures S4A, S4B for the HP chain system at *N* = 4,000 and the patchy particle system at *N* = 500. For each system, we report binodals calculated at three $L_{z}/L_{x}$ ratios. The lowest of these ratios is close to the cubic limit, and it leads to underestimation of the critical temperature. As already pointed out, at $L_{z}/L_{x}$ close to 1, there is very little space for the bulk phase. As the critical temperature is approached, the interface between the dense and bulk phases widens, leaving little space for a fully formed dense phase as well. As a result, the dense-phase density becomes too low whereas the bulk-phase density becomes too high. These opposite errors lead to narrowing of the binodal near the critical point and the underestimation of *T*_c_. In contrast, when $L_{z}/L_{x}$ is within the recommended range of 3–5, or at a higher value as long as the single-slab morphology is sampled at equilibrium for a sufficiently long time, the calculated binodals reach convergence. Further validation of convergence is provided by a comparison of *T*_c_ values at $L_{z}/L_{x}$ ≥ 1.66 and *N* from 1,000 to 10,000 for LJ particles and LJ and HP chains or *N* from 250 to 1,250 for patchy particles (Supplementary Figure S5). The minimum particle number for robust binodal calculation is 4,000 for LJ particles and LJ and HP chains and 500 for patchy particles. *T*_c_ values determined at smaller particle numbers show greater variations. The results for patchy particles also demonstrate that SpiDec works in MC simulations just as well as it does in MD simulations, as shown for LJ particles and LJ and HP chains. We also applied SpiDec in MC simulations of LJ particles. The binodals obtained in MD and MC simulations show close agreement (Supplementary Figure S6A).

We also tested for possible effects of the spring constant on the binodal of the LJ chain system. As shown in Supplementary Figure S7A, the effects are very modest. The binodal remains essentially unchanged when the spring constant is reduced from 75,000 $\varepsilon /\sigma^{2}$ to 750 $\varepsilon /\sigma^{2}$ , and shows a small decrease in *T*_c_ when the spring constant is further reduced to 75 $\varepsilon /\sigma^{2}$ . The spring constant potentially can affect the overall size of the chain, e.g., as measured by the radius of gyration, *R*_g_. However, in the dense phase, chain configurations are largely dictated by inter-chain attraction. The histograms of *R*_g_ are essentially identical when the chain spring constants are 75,000 $\varepsilon /\sigma^{2}$ and 75 $\varepsilon /\sigma^{2}$ , with a mean value of 1.49 $\sigma^{2}$ , which is expanded, due to inter-chain attraction, from a value of 1.28 $\sigma^{2}$ for a freely jointed chain with 10 beads. The domination of inter-chain attraction over intrinsic chain flexibility explains why the chain spring constant has at most a very modest effect on the binodal.

We also tested the effect of the HP chain sequence, specifically the positions of the two P beads. As shown in Supplementary Figure S7B, the positions of the P beads have a significant effect on the binodal. *T*_c_ has a significant increase when the two P beads are moved next to each other. P beads are repulsive to all beads, and the repulsion can be minimized when P beads are clustered together. When the two P beads within a chain are next to each other, P beads are much easier to cluster, and this increased clustering explains the increase in *T*_c_.

### Interfacial tensions of three model systems

The single-slab morphology generated by SpiDec also allows us to calculate the interfacial tension. The results are shown in Figures 5C,D for the LJ particle and LJ chain systems and in Supplementary Figure S4C for the HP chain system, all at *N* = 4,000. Again, as long as *L*_z_/*L*_*x*_ is above 2, convergent results are obtained. We also obtained very similar interfacial tension for the LJ particle system from both MD and MC simulations (Supplementary Figure S6B).

Interfacial tension is a measure of the different extents of intermolecular interactions in the two phases on the opposite sides of an interface. As the temperature approaches *T*_c_, the two phases become more and more similar. Correspondingly the interfacial tension decreases as *T* approaches *T*_c_ and finally vanishes at *T*_c_, where the two phases become identical.

### Application of SpiDec to a peptide system modeled at the all-atom level in explicit solvent

**FIGURE 6:**
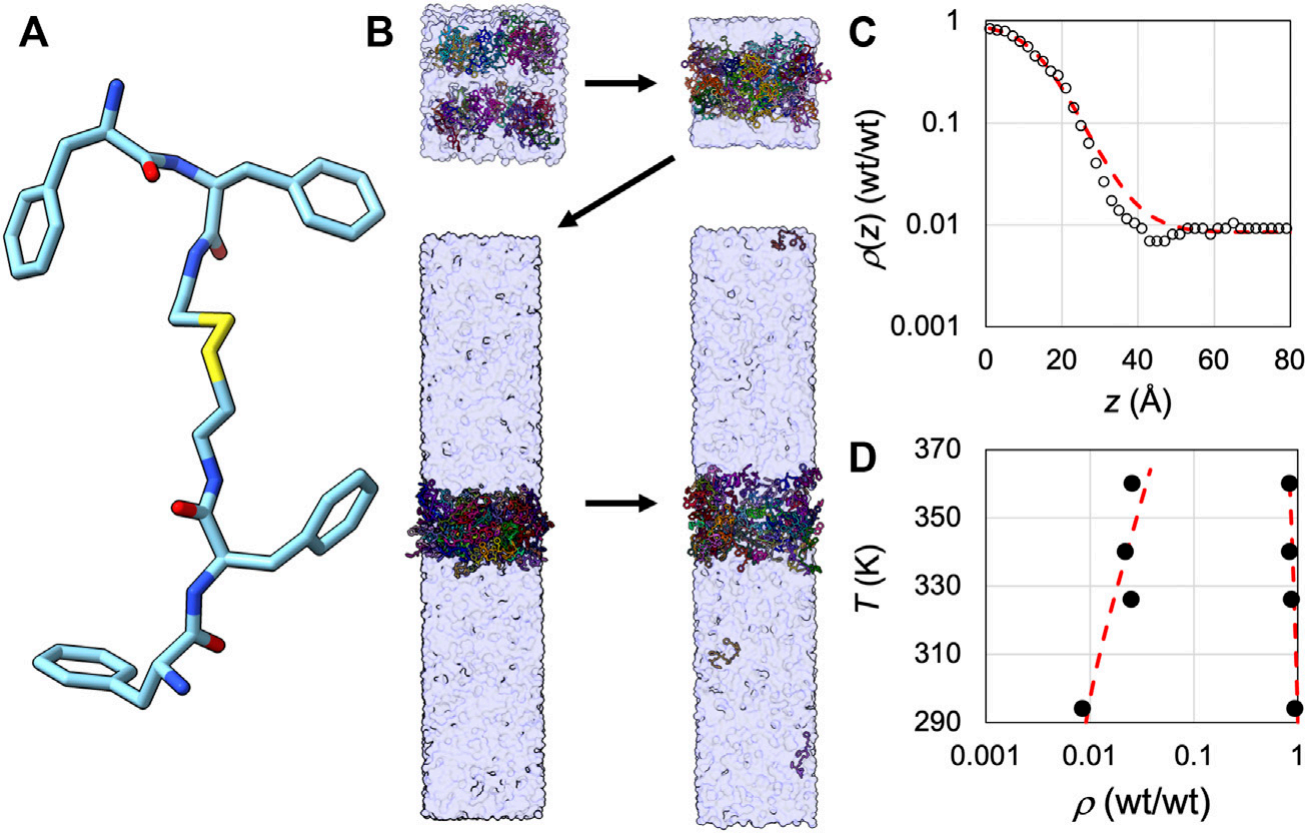
Application of SpiDec to a phase-separating peptide. **(A)** Structure of FFssFF. **(B)** The SpiDec simulation procedure. First, a random, loose configuration condensed into a single slab. Then the slab was solvated into an elongated box and the two phases reached equilibrium. The fourth snapshot shown was taken at 1.065 µs of a 2-µs simulation at 294 K. **(C)** Concentration profile (circles) at 294 K and fit to eq [6] (red curve). Concentrations were calculated by counting copy numbers in 2-Å slices along the *z* direction; the location of each copy was represented by the midpoint of the central disulfide bond. The conversion of concentrations to wt/wt used a molecular weight of 741.5 Da for the peptide and a density of 1 kg/L for water. **(D)** Dilute- and dense-phase concentrations at four temperatures, from 294 K to 360 K. The red curve shows a binodal to guide the eye.

Finally we tested SpiDec on the tetrapeptide FFssFF (Figure 6A) that was recently shown to phase separate (Abbas et al., 2021). Starting from a random, loose configuration solvated in water, 64 copies of the peptide condensed into a single slab (Figure 6B). We subsequently solvated this single slab in an elongated box (*L*_z_/*L*_*x*_ = 5). The copies equilibrated between the dense and bulk phases. We calculated the average concentrations (expressed as wt/wt, i.e., weight of peptide over weight of water) along the *z* axis, and fit the concentration profile to eq [6] to obtain the dense- and bulk-phase concentrations (Figure 6C). At 294 K, the two concentrations are 0.94 wt/wt and 0.0085 wt/wt, respectively. These values are each about 3-fold higher than the experimental counterparts (Abbas et al., 2021), reflecting the need for improved force-field parameterization. We also carried out simulations at 326, 340, and 360 K. At the elevated temperatures, the two densities move toward each other (Figure 6D), as expected for a system showing upper critical solution temperature.

## Discussion

We have characterized the morphologies and timescales of spinodal decomposition in model systems and demonstrated that slab formation *via* spinodal decomposition can be used to calculate the binodal and interfacial tension. At different initial densities in a rectangular simulation box, spinodal decomposition produces distinct dense-phase morphologies, including sphere, cylinder, slab, hollow cylinder, and hollow sphere. The range of initial densities for slab formation is the widest among all the dense-phase morphologies, and this range further widens as *L*_*z*_/*L*_*x*_ increases. At $L_{z}/L_{x}$ above ∼3, multiple slabs emerge from spinodal decomposition. The time for multiple slabs to fuse into a single slab increases rapidly with increasing $L_{z}/L_{x}$ . The optimal $L_{z}/L_{x}$ is thus 3 to 5 for computing the binodal and interfacial tension. Most importantly, we have shown that the SpiDec method is effective both for model systems and for all-atom phase-separating peptides solvated in TIP3P water. In this method, one starts simulations with the system in a homogenous solution, and relies on spinodal decomposition to rapidly bring the system to a two-phase state.

In addition to computing the equilibrium properties (binodal and interfacial tension) of phase separation, SpiDec simulations can be adapted to study dynamic properties of related processes. In particular, the fusion between multiple slabs observed here corresponds to condensate fusion. Therefore SpiDec simulations at large $L_{z}/L_{x}$ can be used to model condensate coarsening, potentially providing a molecular view into the mechanism and kinetics of the coarsening process. Moreover, all the simulations reported above have resulted in complete phase separation, but at lower temperatures (or equivalently, at stronger intermolecular attraction), as expected (Foffi et al., 2005), spinodal decomposition may be arrested, leading to gelation (Supplementary Movie S2). Therefore SpiDec simulations at those conditions provide a means to study condensate gelation.

For the phase-separating tetrapeptide, our simulation results agree qualitatively with experimental data (Abbas et al., 2021), but quantitatively, the computed concentrations in the two phases are each about 3-fold higher than the experimental counterparts. Binodals are exquisitely sensitive to force fields, and now, with SpiDec, we will have the opportunity to use experimental binodals for force-field parameterization of IDPs, leading to accurate modeling of IDPs and their condensate properties.

The advantages of SpiDec can be summarized as follows. Whereas the classical slab method yields only phase equilibrium properties, SpiDec can also be used to study gelation and condensate fusion. For atomistic systems, the implementation of the slab method involved first carrying out a simulation at the coarse-grained level and then mapping a resulting dense slab to the atomistic level (Zheng et al., 2020; Welsh et al., 2022). The mapping is a complicated procedure, and in the subsequent atomistic simulation the protein molecules can easily get trapped in the dense slab. In contrast, as demonstrated here, SpiDec eliminates the need for an initial coarse-grained simulation and directly yields an atomistic system that exchanges between the phases. For systems without any prior knowledge of the binodal, it may be difficult to pick the right initial concentration and therefore one may need to start SpiDec simulations at several initial concentrations. We expect this difficulty to diminish as SpiDec is used on more systems.

## Associated content

### Supporting information

The following Supporting Information is available:

Seven additional figures (Supplementary Figures S1–S7) presenting the dense-phase morphologies of HP chains; the timescales for phase separation and for complete fusion of multiple slabs into a single slab, and illustration of their determination; binodal and interfacial tension of HP chains and binodal of patchy particles; the effects of system size and *L*_*z*_/*L*_*x*_ ratio on the calculated critical temperature; comparison of the binodal and interfacial tension of LJ particles determined by MD and MC simulations; binodals of the LJ chain system with different spring constants and of the HP chain system with different sequences and Supplementary Movie S1 showing the phase separation of LJ particles *via* spinodal decomposition and the fusion of multiple slabs, and Supplementary Movie S2 showing arrested spinodal decomposition leading to gelation of HP chains at *T* = 0.2.

## Data Availability

The raw data supporting the conclusion of this article will be made available by the authors, without undue reservation.

## Abbreviations

HP, hydrophobic-hydrophilic; IDP, intrinsically disordered protein; LJ, Lennard-Jones; MC, Monte Carlo; MD, molecular dynamics; WCA, Weeks-Chandler-Anderson.
