# Supplementary material for: SpiDec: Computing binodals and interfacial tension of biomolecular condensates from simulations of spinodal decomposition
Source: Front Mol Biosci. 2022 Oct 24;9:1021939. doi: 10.3389/fmolb.2022.1021939 (PMC9637972; doi:10.3389/fmolb.2022.1021939)
Supplement: Supplementary file 2 [file DataSheet1.pdf]

SpiDec: Computing Binodals and Interfacial Tension of Biomolecular Condensates From Simulations of Spinodal Decomposition

Konstantinos Mazarakos[†], *Ramesh Prasad*[‡], *and Huan-Xiang Zhou*[†,‡]*

[†]Department of Physics and [‡]Department of Chemistry, University of Illinois at Chicago, Chicago, Illinois 60607, United States

Supporting Information

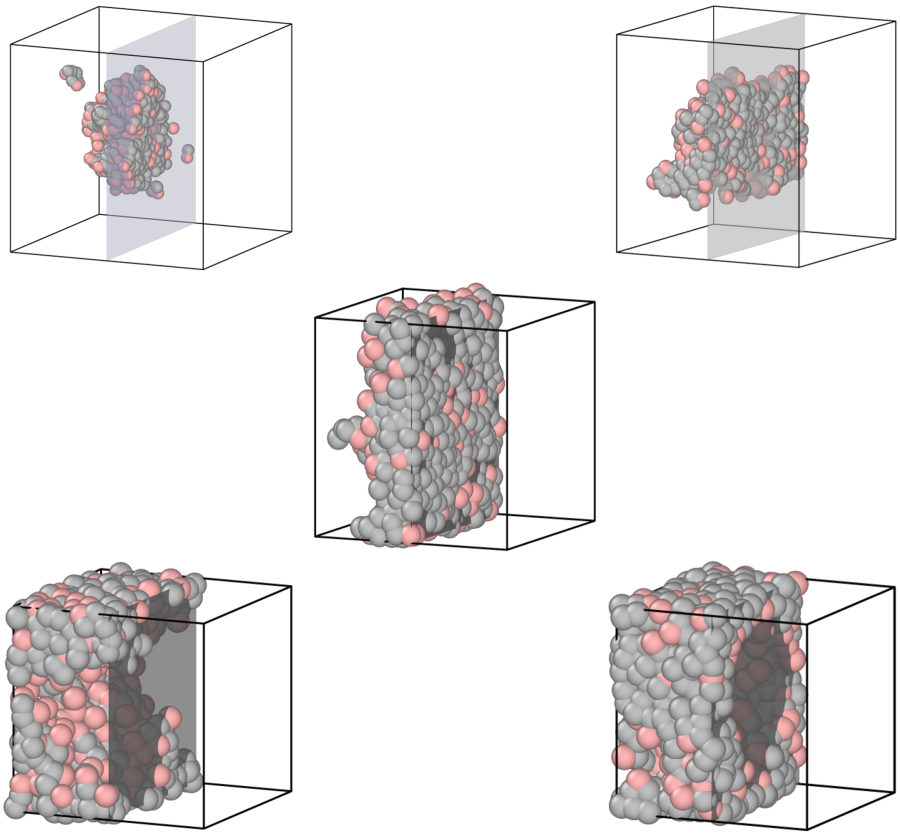

Figure S1. Morphologies of the dense phases of HP chains over a range of initial densities inside the spinodal. 100 chains of 10 beads each were simulated in a cubic box at $T = 1.05$. The dense phase appears as a sphere, cylinder, slab, hollow cylinder, and hollow sphere at ρ_0 = 0.05, 0.1, 0.3, 0.4, and 0.5 respectively. The initial densities were changed by varying the box side lengths, which are shown here not to scale. At each density, the cubic box is cut by a plane (rendered as gray when the background is empty), and only the half behind the cut is displayed.

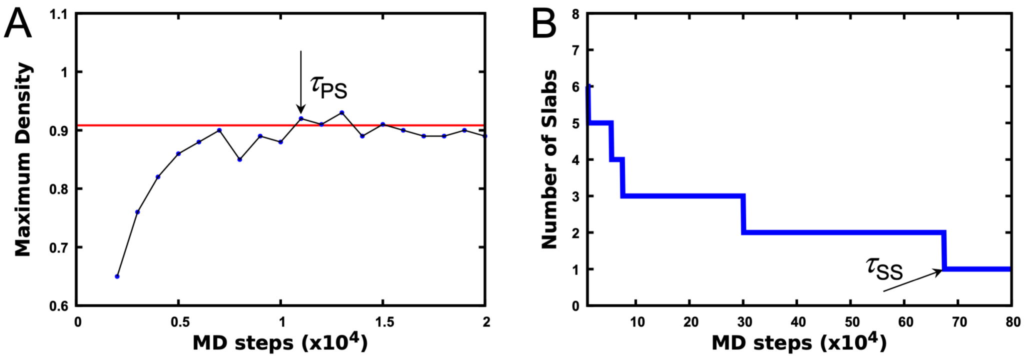

Figure S2. Determination of the timescales for phase separation and for complete fusion of multiple slabs into a single slab, illustrated by the LJ particle system at $N = 4000$, $T = 0.65$, $\rho_0 = 0.3$, and $L_z/L_x = 13.3$. (A) Maximum density within a snapshot, as a function of time. A horizontal line indicates the long-time plateau of the maximum density. An arrow indicates the first snapshot where the maximum density exceeds the long-time plateau, defining the timescale for phase separation. Here the thickness of the slices for density calculation was 1σ, which was used in all the model systems except for HP chains. In that case the thickness was increased to 10σ, because spinodal decomposition produced a domain with a thick, dense core but without flat interfaces; that core would be counted as a slab if the slice thickness were 1σ. (B) Number of slabs as a function of time. An arrow indicates the first snapshot where the number of slabs reaches 1, defining the timescale for complete fusion into a single slab.

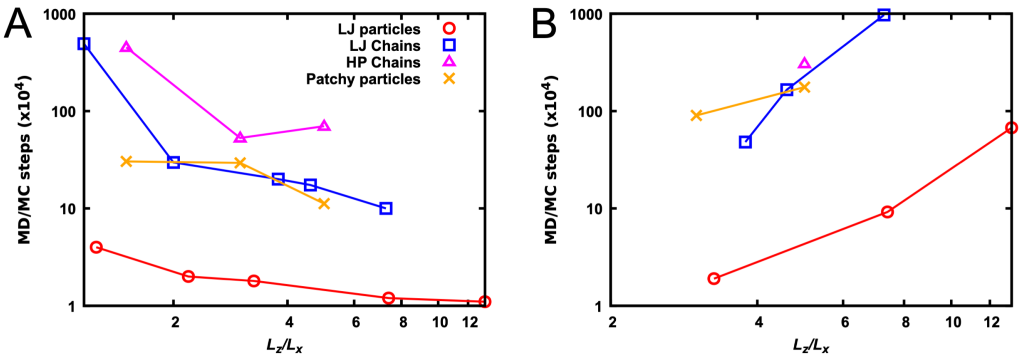

Figure S3. Times for phase separation and for complete fusion into a single slab. (A) Time for slabs for first emerge from spinodal decomposition. (B) Time for the complete fusion of multiple slabs into a single slab. For LJ particles and LJ and HP chains, the particle (bead) numbers are all 4000; the temperatures are 0.65, 1.7, 1.05, respectively; and the initial densities are 0.3, 0.25, 0.25, respectively. The parameters for patchy particles are $N = 500$, $T = 0.61$, and $\rho_0 = 0.36$. Times are shown in units of 10^4 MD or MC steps.

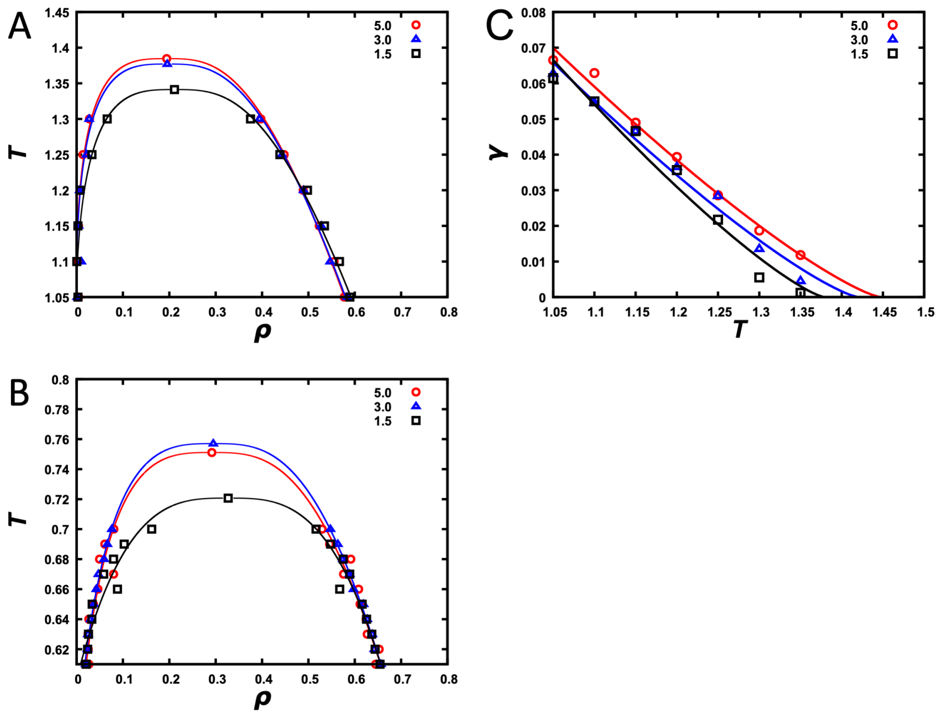

Figure S4. Binodals and interfacial tensions calculated from snapshots with a single slab. (A) Binodals of the HP chain system at $N = 4000$ and $L_z/L_x = 1.5$, 3.0, and 5.0. (B) Binodals of the patchy particle system at $N = 500$ and $L_z/L_x = 1.5$, 3.0, and 5.0. (C) Interfacial tension versus temperature for the HP chain system at the three L_z/L_x ratios.

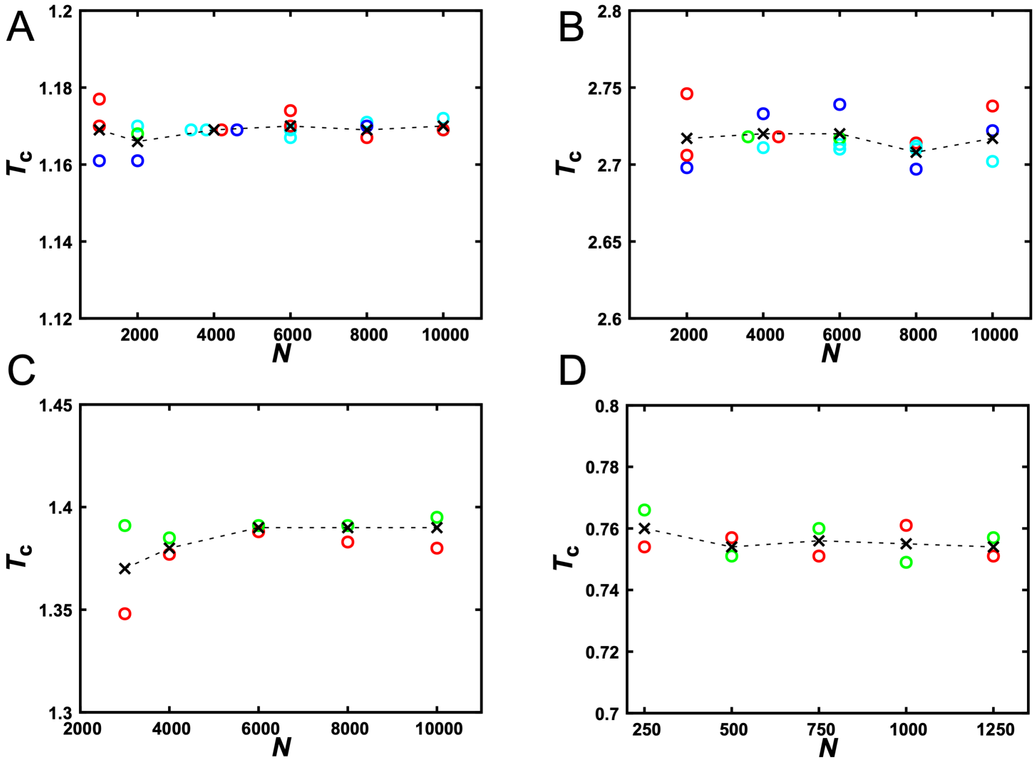

Figure S5. Comparison of critical temperatures obtained at various N values and L_z/L_x ratios. (A) LJ particles. (B) LJ chains. (C) HP chains. (D) Patchy particles. T_c data are displayed in circles with the following color scheme according to L_z/L_x ratios: blue, 1.66-2.5; red, 2.5-4.0; green, 4.0-6.0; and cyan, > 6.0. Crosses show averages T_c values at a given N.

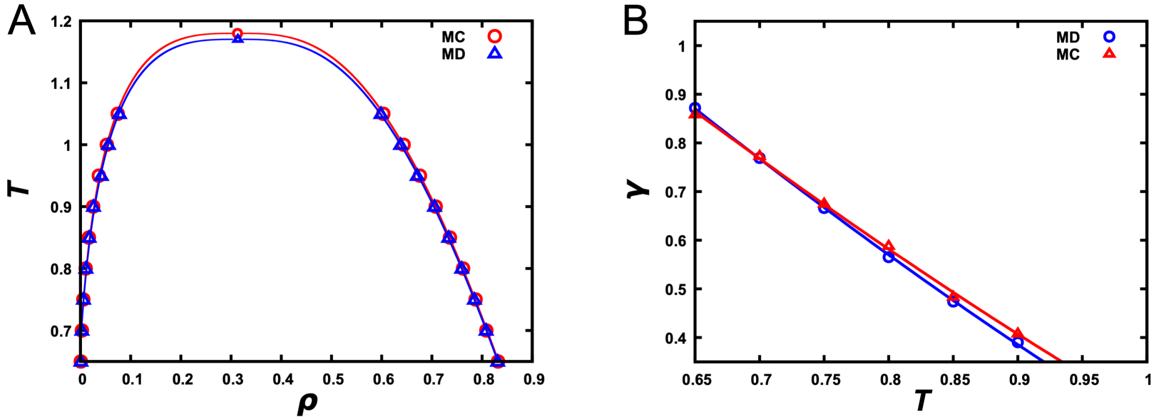

Figure S6. Comparison of binodals and interfacial tensions calculated from MD and MC simulations of LJ particles at $N = 1000$ and $L_z/L_x = 3$. (A) Binodals. (B) Interfacial tensions.

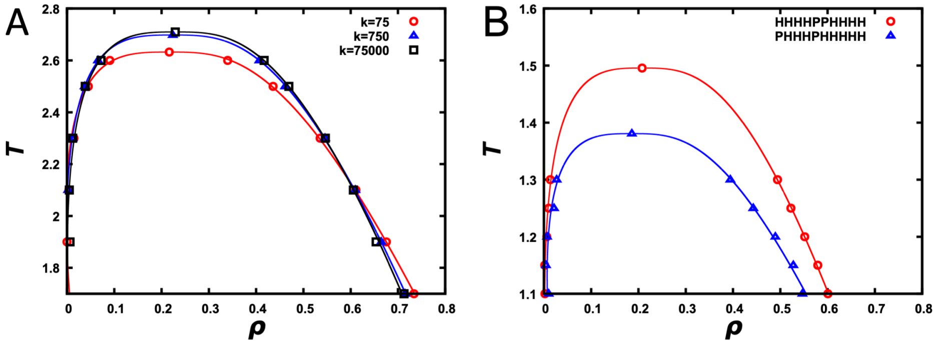

Figure S7. Binodals of the LJ chain and HP chain systems. (A) The LJ chain system at N = 4000 and L_z/L_x = 3.79, with three values for the chain spring constant. (B) The HP chain system at N = 4000 and L_z/L_x = 3.0, with the two P beads at either positions 1 and 5 or positions 5 and 6.

Supporting Movie S1. Phase separation of LJ particles via spinodal decomposition and the fusion of multiple slabs, at $N = 4000$, $T = 0.65$, $\rho_0 = 0.3$, and $L_z/L_x = 13.3$. The entire movie consists of 800 snapshots sampled at intervals of 1000 MD steps. Snapshots 9 and 800 are shown in Fig. 2A.

Supporting Movie S2. Arrested spinodal decomposition and gelation of HP chains, at $N = 4000$, $T = 0.2$, $\rho_0 = 0.25$, and $L_z/L_x = 5$. The entire movie consists of 400 snapshots sampled at intervals of 10000 MD steps.
